# Experience-dependent persistent expression of *zif268* during rest is preserved in the aged dentate gyrus

**DOI:** 10.1186/1471-2202-14-100

**Published:** 2013-09-13

**Authors:** Ali Gheidi, Erin Azzopardi, Allison A Adams, Diano F Marrone

**Affiliations:** 1 Department of Psychology, Wilfrid Laurier University, 75 University Ave W, Waterloo, ON N2L 3C5, Canada; 2 McKnight Brain Institute, University of Arizona, Tucson, AZ 85724, USA

**Keywords:** Fascia dentata, egr1, ngfi-a, Reactivation, Replay, Granule cell, IEG, Hippocampus

## Abstract

**Background:**

Aging is typically accompanied by memory decline and changes in hippocampal function. Among these changes is a decline in the activity of the dentate gyrus (DG) during behavior. Lasting memory, however, is thought to also require recapitulation of recent memory traces during subsequent rest – a phenomenon, termed memory trace reactivation, which is compromised in hippocampal CA1 with progressive age. This process has yet to be assessed in the aged DG, despite its prominent role in age-related memory impairment. Using *zif268* transcription to measure granule cell recruitment, DG activity in adult and aged animals was assessed both during spatial exploration and as animals remained at rest in the home cage in order to detect potential memory-related replay.

**Results:**

Consistent with the observation of memory trace reactivation in DG, the probability that an individual granule cell transcribes *zif268* during rest in the animal’s home cage is increased by recent experience in a novel environment. Surprisingly, a comparable increase was observed in the probability of granule cells in the aged DG expressing *zif268* during rest. Moreover, no significant age-related difference was observed in the number of granule cells expressing *zif268* during rest. Thus, the number and pattern of granule cell expression of *zif268* during rest is preserved in aged animals, despite a significant decline in exploration-related *zif268* expression.

**Conclusions:**

These data lead to the hypothesis that the input the aged DG receives from backprojections from CA3 (the region widely hypothesized to mediate reactivation) remains functionally intact despite loss of innervation from the perforant path.

## Background

Aging is often accompanied by memory decline, even in the absence of pathology [1,2]. Consistent with the well-documented role of the hippocampus in memory formation, aged animals show robust memory deficits, and demonstrate major changes in hippocampal function during spatial processing. One of the most dramatic of these changes is a significant decrease in the activity of the dentate gyrus (DG). In fact, several researchers have proposed that the DG represents one of the major targets of age-related hippocampal dysfunction [1,2].

Lasting memory, however, is thought to require the continual consolidation of encoded representations into more permanent states. This is hypothesized to involve coordinated recapitulation of memory traces while the hippocampus is “off-line” (i.e., not processing external stimuli), a phenomenon termed memory trace reactivation. In fact, the replay of behaviorally-elicited patterns of network spiking activity during subsequent resting states (i.e., both sleep and quiet wakefulness) has been repeatedly observed in multiple brain regions (for review, see [3,4]). Moreover, it has recently been shown that memory trace reactivation in CA1 becomes less reliable with progressive age and this deficit correlates with declines in hippocampus-dependent memory [5]. The fidelity of reactivation has yet to be assessed in the aged DG, however, despite the fact that this region participates in reactivation [6] and plays a critical role in age-related memory impairment [7].

This lack of data may largely be due to technical challenges posed by the sparsity of activity in granule cells [8]. These challenges are further magnified by the fact that DG activity is further reduced in aged animals [7]. These obstacles, however, can be overcome by the use of histological techniques that are capable of recording the activity of thousands of cells during both spatial behavior [9] and subsequent replay [10,11]. Such techniques may be conducted using *zif268* (also named *egr1*), an immediate-early gene that is critical for enduring memory and synaptic plasticity [12] and that is thought to label cells expressing place fields [9-11]. This is because the transcription of *zif268* provides estimates of granule cell activity [13,14] that are similar to place cell recordings during spatial navigation in comparable conditions [15-17].

Using *zif268*, granule cell activity was assessed in both adult and aged, memory-impaired animals during spatial processing and adjacent periods of rest in the home cage. Observations of *zif268* transcription during rest in the home cage in the same granule cells that transcribed *zif268* during previous spatial navigation provide an estimate of the proportion of the granule cell population engaging in replay of recent spiking activity. Comparing these estimates in adult and aged animals can address whether this process is compromised with progressive age in the DG, consistent with previous observations in hippocampal CA1.

## Methods

### Experiment 1: experience-dependence of DG zif268 expression

#### Subjects

Twenty male Fischer 344 rats (Harlan Sprague Dawley, Indianapolis, IN) aged 8–12 months were used in this experiment. All rats were individually housed on a 12:12 hour light cycle with *ad lib* access to food and water. Prior to behavioral testing, all animals were handled 15 min/day for 10 days to habituate them to the general handling procedure. The procedures described below were carried out at least 2 hours after the commencement of the dark cycle. All procedures were approved by the animal care committee of Wilfrid Laurier University and were carried out in accordance with the guidelines of the Canadian Council on Animal Care.

#### Behavioral procedures

Spatial exploration was conducted as previously described [9]. Rats were divided into 2 behavioral groups. Animals in both of these groups were exposed to a novel environment consisting of an open field (50 × 76 cm) with three black walls and one white wall, each 20 cm high. The open field was divided into 9 equal grids, and during exploration, rats were randomly placed within a different grid every 15 seconds for 5 minutes in order to ensure all aspects of the environment were sampled equally. Although animals were free to move around the environment during the 15 seconds between placements, this short interval results in very little variation in exploratory behavior. One group (immediate; n = 6) was killed, as described below, immediately following this exploration. A second group (delay; n = 7) was returned to their home cage following the exploration and remained there undisturbed for 25 min before being removed and killed. This provides an assessment of the transcription of *zif268* induced by spatial navigation, as well as *zif268* transcription occurring during rest in the home cage during a comparable period of time approximately 30 minutes before (in the case of the immediate group) or approximately 30 minutes after (in the case of the delay group) the behavior. Animals were not monitored during their resting periods within the home cage, and as a result the state of the animal while in the home cage (i.e., quiet wakefulness vs. slow-wave or paradoxical sleep) cannot be confirmed. Previous multi-unit recordings, however, show that reactivation can be observed in any of these states [3,4]. The environments were cleaned thoroughly with 2% acetic acid between subjects.

An additional caged control group (n = 7) was killed directly from the home cage, without any behavioral handling as a negative control in order to establish baseline levels of *zif268* expression.

### Experiment 2: the effect of aging on zif268 recapitulation

#### Subjects

This experiment included 12 adult (aged 9–12 months) and 12 aged (23–26 months) male Fischer 344 rats (Harlan Sprague Dawley, Indianapolis, IN). All rats were individually housed on a 12:12 hour light cycle with *ad lib* access to food and water. All procedures were carried out at least 2 hours after the commencement of the dark cycle.

#### Behavioral procedures

All animals were handled 15 min/day for 10 days to habituate them to general handling. All animals’ spatial learning abilities were then assessed using the Morris swim task, as previously described [13]. A 1.83 m diameter circular watermaze was located in a well-lit room containing prominent local and distal visual cues and filled with water at 25 ± 1°C made opaque with tempura paint (Scholar’s Choice, London, ON). Each animal’s location was tracked using an overhead video camera connected to Any-maze tracking software (Stoelting, Kiel, WI).

The rats first received 6 spatial learning trials per day for 4 days. At the beginning of each trial, rats were placed into the water, facing the maze wall, at one of four start positions (i.e., North, West, South, and East). Rats could escape by swimming to a 12 cm diameter platform ~1 cm below the surface of the water. If an animal failed to reach the platform within 120 s, it was guided there by the experimenter. Every trial ended with the rat sitting on the platform for 30 s. Following spatial training, a probe trial in the absence of the platform tested retention of the platform location. This was followed by 12 visible platform trials with the platform marked and clearly visible above the waterline, to ensure that age-related differences in spatial trials are not due to deficits visual acuity or motor ability. Any aged animals performing outside the range of adult animals in the visible platform trials were excluded from behavioral analysis.

Seven days after the Morris swim task was completed, passive spatial exploration was conducted as described in experiment 1. This passive exploration manipulation is particularly important for age comparisons, since aged rats will navigate considerably less than adults if allowed to navigate freely. Six adult and 6 aged animals were exposed to the novel environment and returned to their home cages for 25 min before being removed and killed. The remaining 6 adult and 6 aged caged control animals were killed directly from the home cage as negative controls.

### Methods common to both experiments

#### Tissue preparation

At the end of behavioral testing, the rats were anesthetized by isoflurane, decapitated, and their brains were rapidly removed and flash frozen in isopentane submerged in ethanol/dry ice. The frozen brains were then grouped, using optimal cutting temperature (OCT) medium (Fisher Scientific, Whitby, ON) to include tissue from all behavioral groups in a block. Coronal sections (20 μm-thick) were obtained from each block and collected on Superfrost Plus slides (VWR Scientific). Because these brains were sectioned together in a single block of OCT, these sections being compared across the block were anatomically matched.

Fluorescent *in situ* hybridization was then performed on these slides as previously described [9,13]. Briefly, digoxigenin-labeled *zif268* antisense riboprobes were synthesized using a transcription kit (Ambion, Austin, TX) and a nucleotide mix containing digoxigenin-labeled UTP (Roche Applied Science, Montreal, PQ), denatured, and hybridized with the tissue overnight at 56°C. Following post-hybridization washes of gradually increasing stringency and 2% H_2_O_2_ to quench endogenous peroxidase, the riboprobe was detected with anti-digoxigenin-HRP conjugate (Roche) and a cyanine-3 substrate kit (PerkinElmer Life Sciences, Boston, MA). Slides were counterstained with DAPI (Sigma), coated with Vectashield anti-fade media (Vector Labs, Burlington, ON), and sealed.

#### Imaging

**Figure 1:**
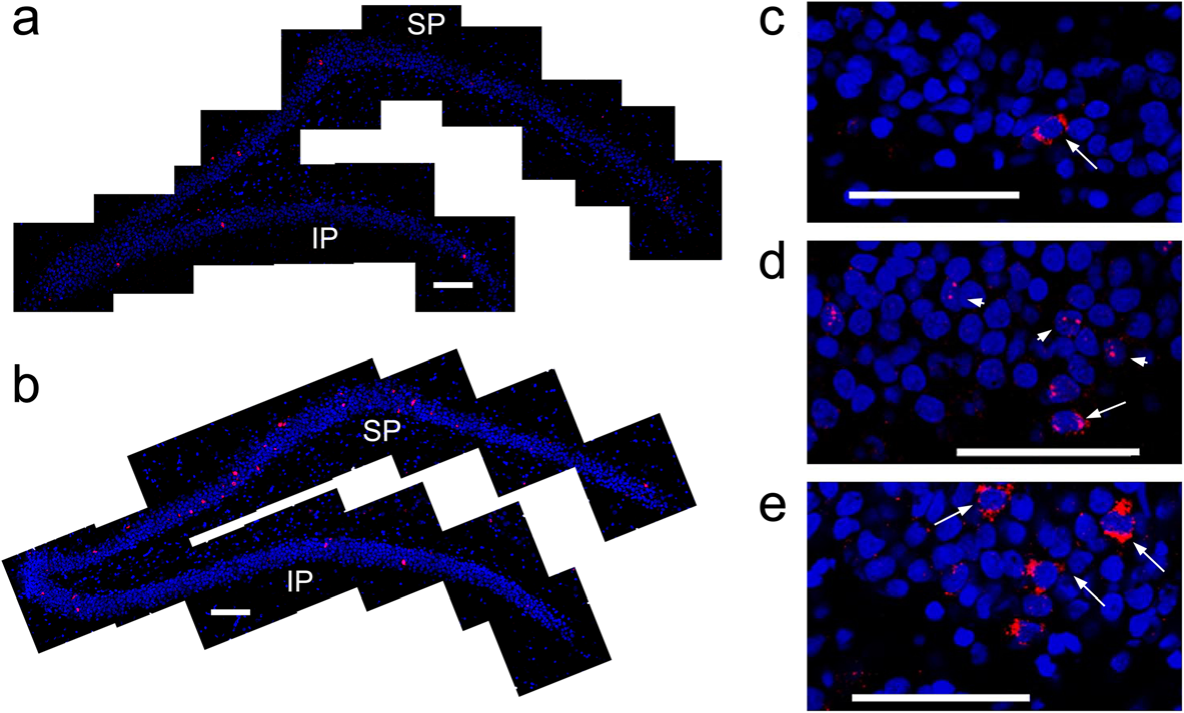
**The pattern of *zif268 *expression in the dentate gyrus (DG) is altered by recent experience.** Representative montages of confocal images taken across the DG (scale bar = 200 μm) demonstrate that the number of granule cells expressing *zif268* in caged control animals **(a)** taken directly from their home cages is very low (<1%) in both blades of the DG, while spatial exploration **(b)** induces a robust increase in *zif268* transcription localized primarily in the suprapyramidal (SP) blade and not the infrapyramidal (IP) blade. Higher magnification confocal micrographs of the suprapyramidal blade (scale bar = 60 μm) are also shown to illustrate the differences in the pattern of *zif268* transcription in the three behavioural conditions implemented in the current study. Relative to caged controls **(c)**, the DG of animals killed immediately following spatial exploration (**d**, immediate) show significantly more granule cells (~5%) expressing *zif268* within the nucleus (short arrow). Animals killed 25 min after exploration (**e**, delay) show comparable levels of *zif268* expression to the immediate group, but the majority of mRNA is within the cytoplasm of granule cells (long arrow). Granule cell nuclei are counterstained with DAPI (blue) and *zif268* is labeled with Cy3 (red).

As described previously [9,13], z-stacks (optical thickness: 1.1 μm, interval: 0.7 μm) throughout the thickness (~20 μm) of each blade of the dorsal DG (−3.2 to −3.8 mm from Bregma [18]) were acquired from 5–6 slides per animal (Figure 1). Images were collected using an Olympus FV1000 laser scanning confocal microscope at 40× with photomultiplier tube assignments, confocal aperture size, and contrast values kept constant for each slide. Image analysis was then conducted using Metamorph 7 (Molecular Devices, Downingtown, PA).

**Figure 2:**
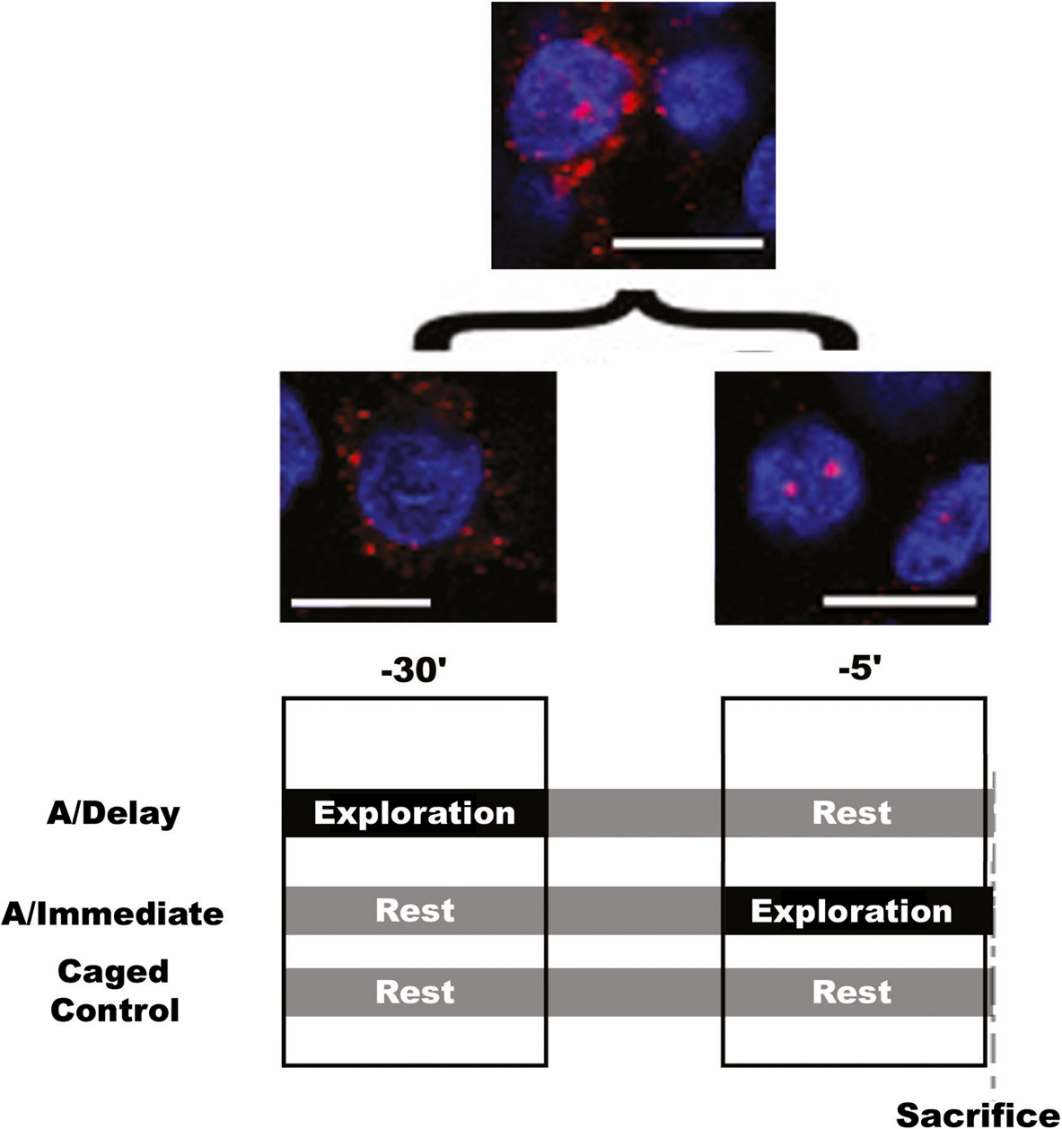
**Representative confocal images (scale bar = 10 μm) depict the compartmental expression of IEGs (above) and their temporal relationship to the experiences of the animals used in the experiments described here (below).** Cells (counterstained with DAPI, blue) that exhibit high frequency firing of the type that characterize place fields begin to express IEGs such as *zif268* (red) within minutes. When this activity occurs 30 minutes prior to the animal being killed (during the time in which both caged control and immediate animals are at rest in the home cage, while delay animals are exploring a novel environment), the transcripts migrate to the cytoplasm where they are apparent as a diffuse cloud (bottom left). In contrast, when this activity occurs within 5 minutes of the animal being killed (during the time in which both caged control and delay animals are at rest in the home cage and immediate animals are exploring a novel environment), the transcripts are visible as bright foci within the nucleus (bottom right). Cells active during both time periods will express *zif268* within both cellular compartments (top).

Granule cells were counted by the optical dissector within the median 20% of planes in each confocal stack and were classified into four groups based on the pattern of *zif268* expression: (1) *zif268*-negative, (2) *zif268*-positive in the nucleus, (3) *zif268*-positive in the surrounding cytoplasm, or (4) expressing *zif268* in both the nucleus and cytoplasm (Figure 2). Because the localization of IEGs within a neuron provides a marker of the approximate time at which each neuron was engaged in transcription [9], these gene expression profiles indicate the number of cells active during each of two distinct epochs, occurring ~30 minutes prior to sacrifice of the animal (i.e., epoch 1) and within the 5 minutes immediately prior to sacrifice of the animal (i.e., epoch 2). Note, however, that the total number of cells expressing zif268 within a specific compartment does not correspond precisely to the number of cells active in at a given time. For instance, cells in both categories (3) and (4) above would correspond to the total number of cells active during epoch 1, and both categories (2) and (4) would correspond to the total number of cells active during epoch 2. That is, all cells expressing cytoplasmic *zif268* were counted as active during epoch 1 whether or not they also expressed *zif268* within the nucleus, and all cells expressing nuclear *zif268* were counted as active during epoch 2 whether or not they also expressed *zif268* within the cytoplasm. For illustrative purposes, the proportion of cells expressing *zif268* within each individual cellular compartment is also provided in all figures.

Group differences in proportion of cells transcribing *zif268* during epoch 1, epoch2, or during both epochs (i.e., category 4 above) were analyzed with ANOVA, using condition (i.e., immediate, delay, and caged control) and region (i.e., infrapyramidal or suprapyramidal blade) as factors, followed by Tukey’s HSD *post hoc* tests. To assess whether *zif268* transcription in individual granule cells during rest is predicted by those cells transcribing *zif268* during exploration, the same methods were used that have been applied previously to other hippocampal and cortical regions [10,11]. Briefly, an estimate of the overlap in transcription expected by random chance was generated for each animal as the product of the proportion of cells active during epoch 1 and those active during epoch 2. For instance, if within an individual animal 4% of granule cells transcribed *zif268* during exploration and 1% of cells transcribed *zif268* during rest, the expected random overlap between these two active populations would be 0.4% (0.04 × 0.01 = 0.004). The estimated overlap based on random chance in a given animal and the overlap observed in that animal (i.e., category 4 above) was then compared using a paired *t*-test.

The statistical comparisons in experiment 1 were derived from a count of 13,168 granule cells from the suprapyramidal blade of the DG and 9,414 granule cells from the infrapyramidal blade of the DG, while experiment 2 contained counts from 15,247 suprapyramidal granule cells.

## Results

### Experiment 1: experience-dependence of DG zif268 expression

#### Spatial exploration-induced zif268 transcription

**Figure 3:**
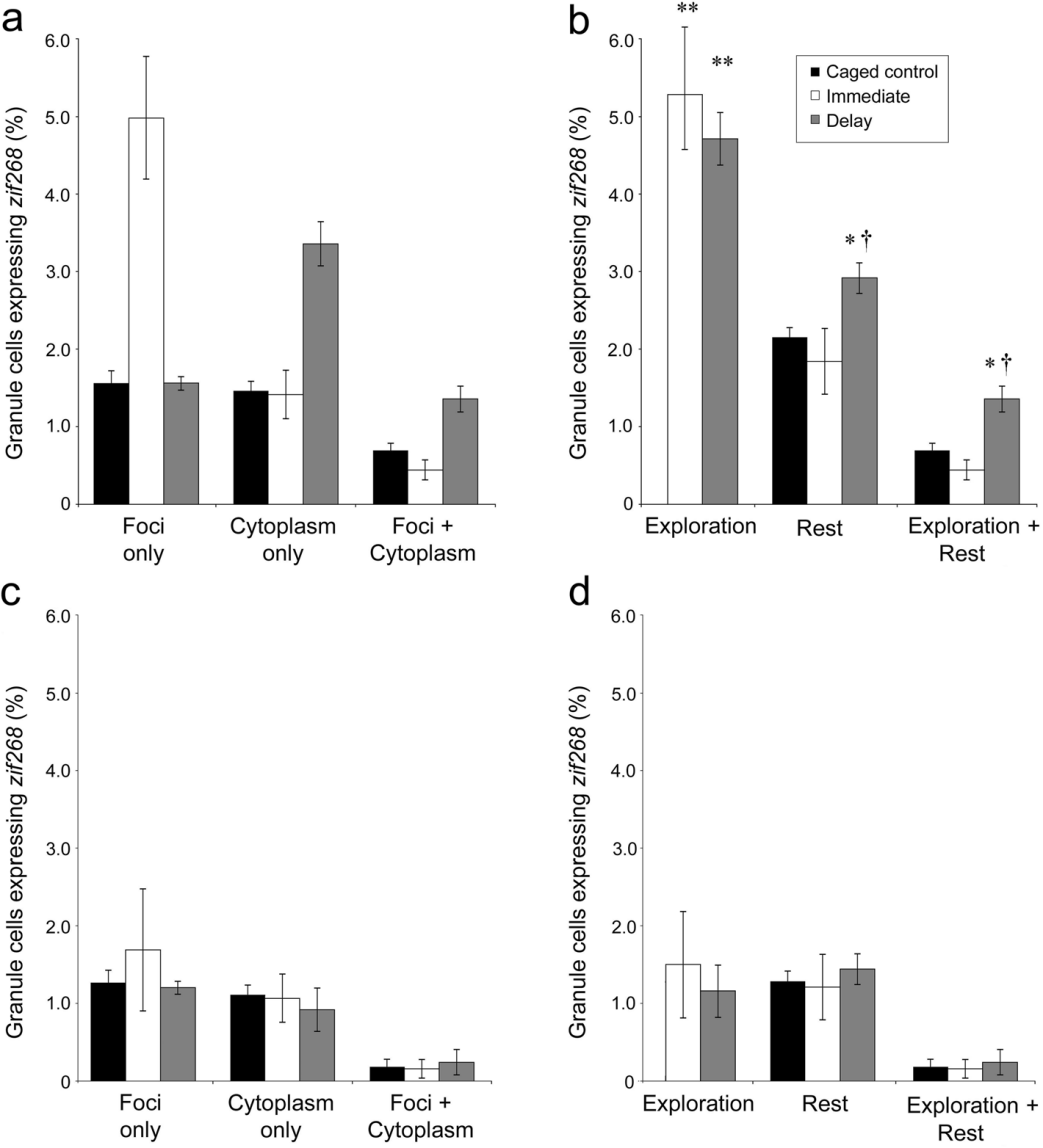
**Behavior-induced *zif268 *expression within the DG. (a)** In the suprapyramidal blade, exploration of a novel environment induced *zif268* in a comparable number of granule cells in both the immediate (white) and delay (grey) groups that was significantly greater than *zif268* expression in caged controls (black). **(b)** While the number of granule cells expressing *zif268* during an episode of rest (i.e., cells transcribing *zif268* while animals were in the home cage would correspond to cells expressing cytoplasmic *zif268* in the immediate group and all cells expressing nuclear *zif268* in the delay group, see Methods) that precedes spatial exploration (immediate) is equivalent to caged controls, significantly more granule cells express *zif268* during a rest episode that follows spatial exploration (delay). Note that *zif268* expression in caged control animals is depicted solely in the “rest” category since these animals were never removed from the home cage and thus their only experience was comparable to the rest period of both immediate and delay animals. In addition, significantly more *zif268* expression occurred in the same cells that were active during exploration (exploration + rest). In the infrapyramidal blade, exploration of a novel environment did not significantly alter the number of granule cells expressing *zif268***(c)** within any cellular compartment or **(d)** during either epoch (all data are mean ± SEM; *p < 0.05, **p < 0.01 vs. CCs; † p < 0.05, immediate vs. delay).

When the number of cells engaged in *zif268* transcription during exploration (i.e., the total number of active cells, calculated as described in the methods, in epoch 1 for delay animals and epoch 2 for immediate animals, see Figure 2) or during rest (i.e., the total number of active cells in epoch 2 for delay animals and epoch 1 for immediate animals) are quantified, analysis reveals that previous experience in a novel environment increases the number of cells transcribing *zif268* during rest (main effect of condition: F_2,34_ = 11.08; p < 0.001). In caged control animals, *zif268* is consistently expressed in ~1.5% of granule cells across the DG (Figure 3). When animals explore a novel environment, however, this proportion is significantly increased to ~5% of DG granule cells. This increase in transcription, however, is restricted largely to the suprapyramidal blade (Figure 3a), as demonstrated by significant main effect of region (F_1,34_ = 56.31; p < 0.001) and a significant region by condition interaction (F_2,34_ = 6.83; p = 0.003). Post-hoc tests confirm that while *zif268* expression occurs in significantly more suprapyramidal granule cells in both immediate (p = 0.002) and delay (p = 0.009) animals relative to caged controls, these significant differences are not apparent in the infrapyramidal blade (immediate: p = 0.202; delay: p = 0.208; Figure 3b). These data are consistent with several previous reports (e.g., [8,19-21]) showing little IEG expression in the infrapyramidal blade following spatial behavior. The key comparison for the current data, however, lies in the expression of *zif268* in granule cells during rest, and the relationship this transcription pattern may have with the population of cells recruited during spatial exploration.

#### Previous experience alters zif268 transcription during rest

Analysis of rest episodes (Figure 3) reveals a significant main effect of both region (F_1,34_ = 27.41; p < 0.001) and condition (F_2,34_ = 4.563; p = 0.02) on *zif268* expression during rest, with no significant interaction (F_2,34_ = 1.27; p = 0.29). Post-hoc tests show that, while the number of granule cells transcribing *zif268* during rest in immediate animals is comparable to caged controls (suprapyramidal blade: p = 0.54; infrapyramidal blade: p = 0.96), delay animals show a significant increase in the number of granule cell transcribing *zif268*, restricted to the suprapyramidal blade (suprapyramidal blade: p = 0.03; infrapyramidal blade: 0.63). This increase in transcription differs substantially from CA1 under comparable conditions [10,11]. The critical test to determine if the *zif268* expression can be observed in a pattern that is consistent with memory trace reactivation in this region, however, is not tied to the number of active cells. If, as hypothesized, *zif268* transcription during rest is driven by the replay of the patterns of spikes induced by recent behavior, transcription during rest should predominantly occur in the same cells that were active during previous spatial exploration.

#### Repeated zif268 transcription in common cell populations

**Figure 4:**
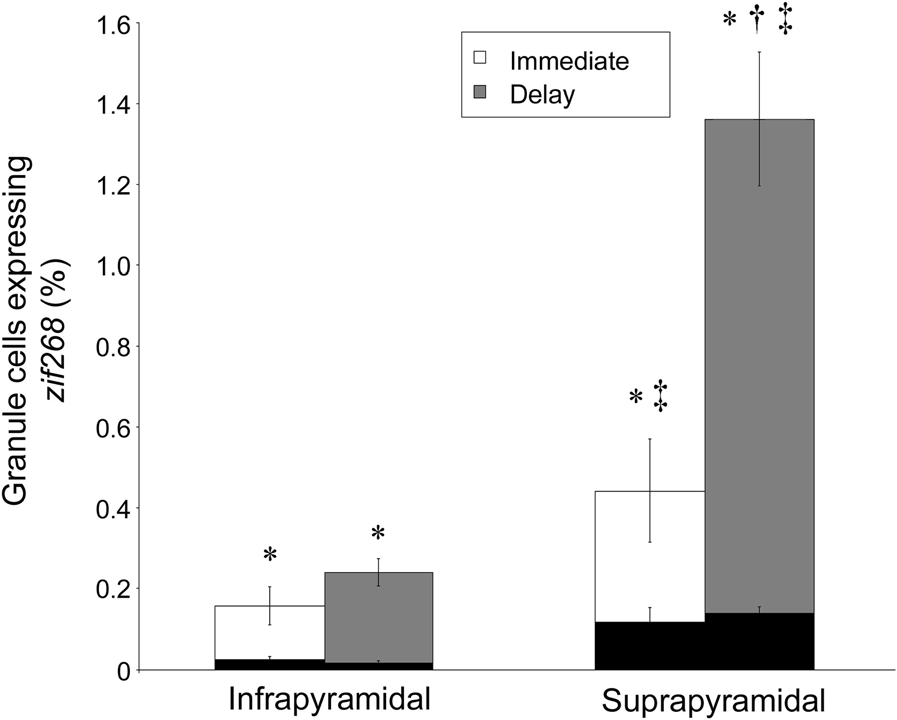
**The behaviorally-induced pattern of *zif268 *transcription is recapitulated during subsequent rest in the DG.** In both immediate (white) and delay (grey) conditions, the percentage of granule cells transcribing *zif268* during both exploration and rest (i.e., expressing *zif268* within both the nucleus and surrounding cytoplasm) is greater than expected by random chance (black), although significantly more cells are active during both rest and exploration in the delay condition in the suprapyramidal blade (all data are mean ± SEM; *p < 0.01 vs. random chance; † p < 0. 01 immediate vs. delay, ‡ p < 0.05 infrapyramidal vs. suprapyramidal blades).

Analysis of the number of granule cells transcribing *zif268* during both exploration and rest episodes shows that transcription in the DG during rest is not random. Using the ability of the catFISH technique to provide a histological record of the activity of a single granule cell at 2 time points demonstrates that the same cells repeatedly transcribe *zif268*. A proportion of this repeated activity, however, is not experience-dependent. That is, the probability that the same granule cell transcribed *zif268* during both exploration and rest episodes was significantly greater than expected by random chance (Figure 4) in all groups: caged control (suprapyramidal blade: *t*_5_ = 6.52; p = 0.001; infrapyramidal blade: *t*_6_ = 3.56; p = 0.01), immediate (suprapyramidal blade: *t*_5_ = 3.40; p = 0.02; infrapyramidal blade: *t*_5_ = 3.12; p = 0.03) and delay (suprapyramidal blade: *t*_6_ = 8.08; p < 0.001; infrapyramidal blade: *t*_6_ = 7.69; p < 0.001) animals. These data are consistent with multi-unit recordings of the DG in freely-moving animals [6,16,17] indicating that even across diverse behavioral experiences, the same population of granule cells tends to fire repeatedly across multiple experiences. Despite this ‘basal’ correlation across multiple episodes, it is still possible to address whether the probability of repeated *zif268* transcription within the same granule cells is further increased by spatial experience.

#### Increased probability of zif268 transcription in granule cells recruited by recent behavior

The order of behavioral experience further altered the proportion of granule cells responding to exploration that also transcribed *zif268* during a previous rest (main effect of condition: F_2,34_ = 17.50; p < 0.001). However, this experience affected the blades of the DG differently (main effect of region: F_1,34_ = 1.27; p = 0.29; region by condition interaction: F_2,34_ = 5.15; p = 0.01). That is, granule cells in the delay group had a significantly higher probability of expressing *zif268* during both exploration and rest in the suprapyramidal blade. In contrast, no difference is seen among granule cells in the infrapyramidal blade based on the order of experience (Figure 4). These findings are consistent with the hypothesis that *zif268* transcription is driven the recapitulation of recent activity patterns, and shows that it is largely the suprapyramidal blade that participates in this process.

### Experiment 2: the effect of aging on zif268 recapitulation

#### Aged animals have impaired spatial learning

Consistent with previous reports (e.g., [13,22-24]), aged animals showed a deficit in spatial, but not cued, trials of the Morris swim task (Figure 4). All rats swam progressively shorter path lengths over trials, creating a significant effect of training day (F_3,66_ = 5.51; p = 0.002). Although no main effect of age was observed on path length (F_1,22_ = 0.99; p = 0.33), a significant age by training day interaction was apparent (F_3,66_ = 3.53; p = 0.02). *Post hoc* analyses confirmed that age-related differences grew progressively larger throughout training, reaching significance on training day 4 (day 1: p = 0.34; day 2: p = 0.82; day 3: p = 0.14; day 4: p = 0.02). These data confirm that the aged rats in the present study had impaired spatial memory.

**Figure 5:**
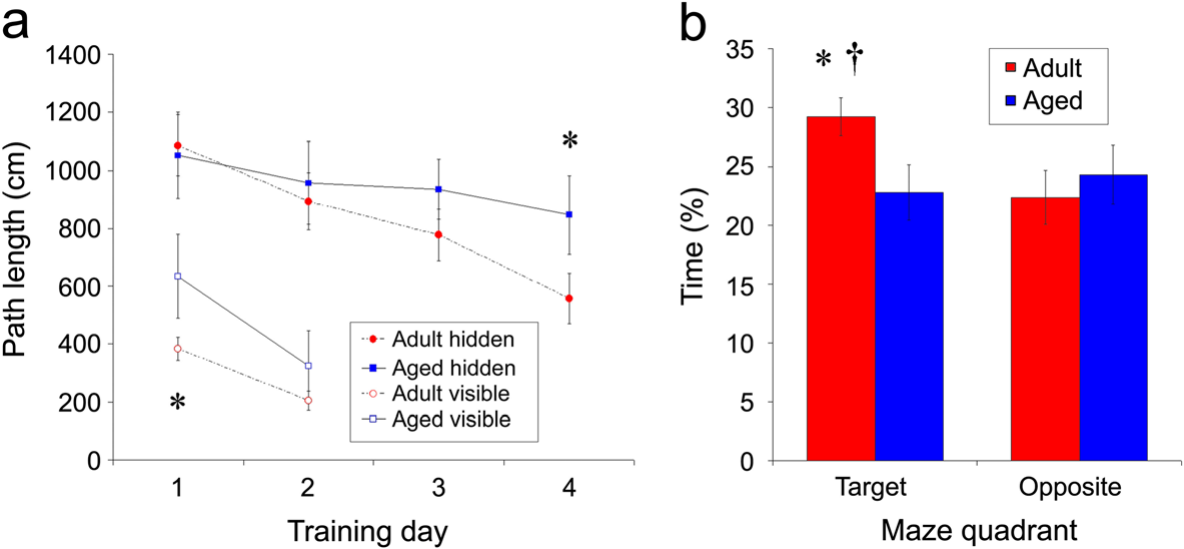
**Aged rats are impaired in the Morris swim task.** Analysis of path lengths **(a)** shows that when the platform was hidden, adult rats (black circle) swam shorter paths to reach the hidden platform than did the aged rats (black square), and this difference was significant on day 4 of training. During trials in which the platform was visible, adult rats (open circle) and aged rats (open square) had comparable path lengths. During the probe trial **(b)**, adult rats (white) spent significantly more time than aged rats (grey) in the quadrant that previously held the platform (target), but not the opposite quadrant (all data are mean ± SEM; *p < 0.05; **p < 0.01, adult vs. aged animals).

During visible platform trials, both adult and aged rats showed significant learning across the 2 days of training (main effect of training day: F_1,22_ = 5.70; p = 0.03). A main effect of age was also observed (F_1,22_ = 8.20; p = 0.01), as well as a significant interaction between age and training day (F_1,22_ = 4.76; p = 0.04). Although *post-hoc* analyses indicate that aged animals took significantly longer paths in order to reach the visible platform on day 1 (p = 0.02), these differences were no longer apparent on day 2 (p = 0.17), demonstrating that, given sufficient training, aged animals were able to consistently and efficiently locate the visible platform. These data strongly suggest that the age-related differences in spatial trials cannot be accounted for solely by deficits in visual acuity or motor ability (Figure 5a).

A consistent age-related deficit in spatial memory was also observed during the probe trial (Figure 5b). Adult rats swam a greater distance in the target platform quadrant (F_1,22_ = 4.95; p = 0.04) than aged rats. Similarly, adult rats spent a significantly greater proportion of time in the quadrant that previously held the escape platform than the opposite quadrant (t_11_ = 3.84; p = 0.003), while aged rats did not (t_11_ = 0.57; p = 0.58).

#### Age-related decrease in behaviorally-induced zif268 transcription

**Figure 6:**
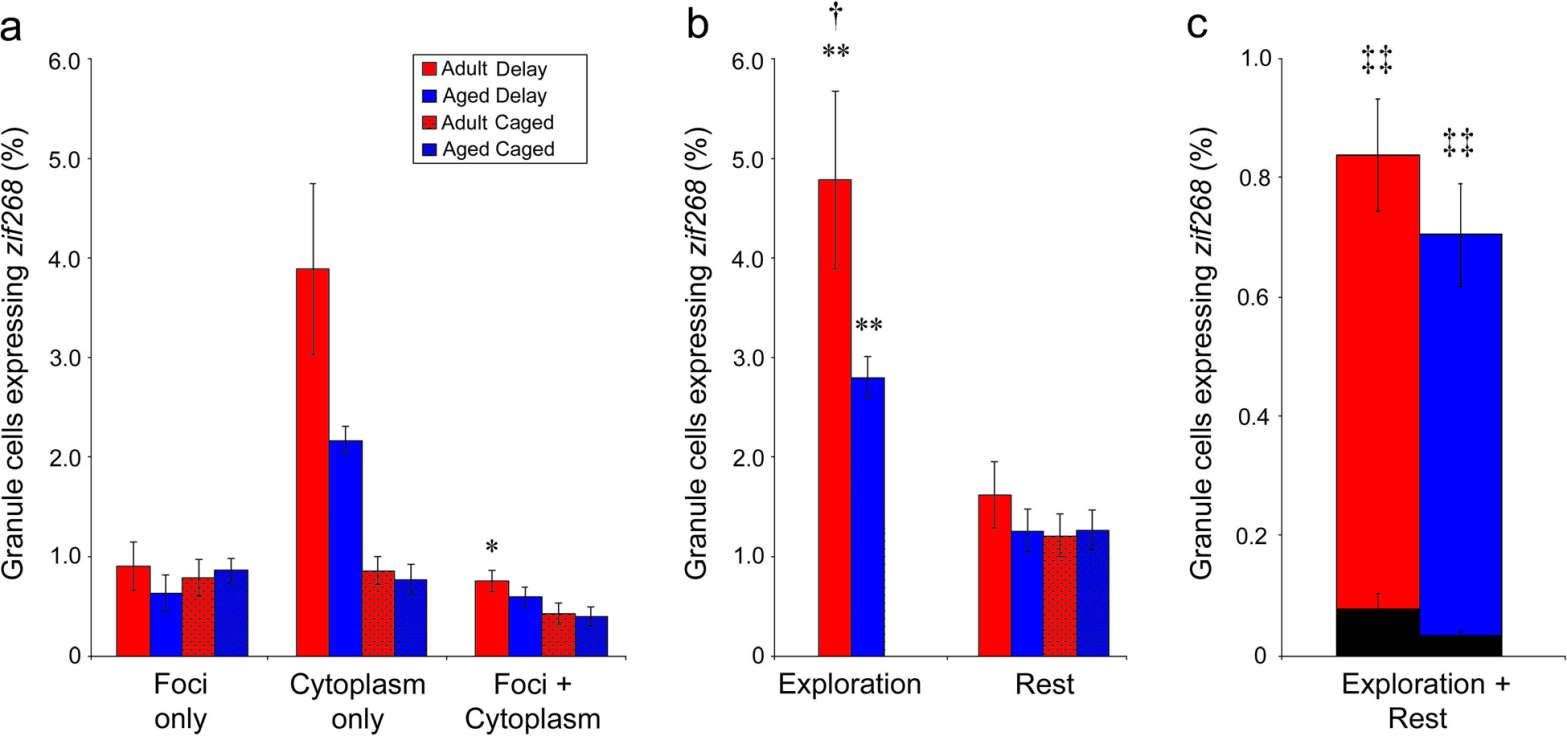
**The expression of *zif268 *in the suprapyramidal DG is attenuated with age during exploration but not rest. (a)** Compartmental expression of *zif268* is depicted for adult (red) and aged (blue) animals that explored a novel environment and those that remained within the home cage (hatched). When *zif268* expression in adult and aged animals is compared during each epoch **(b)** a dramatic decline is apparent in the number of granule cells transcribing *zif268* during exploration in aged animals. During the resting period immediately prior to sacrifice, however, no significant difference is seen in the number of *zif268+* granule cells. Note that *zif268* expression in caged control animals is depicted solely in the “rest” category since these animals were never removed from the home cage and thus their only experience was comparable to the rest period of both adult and aged delay animals. When the percentage of granule cells expressing *zif268* during both epochs (exploration + rest) is analyzed relative to probability of co-expression based on chance **(c)** a significantly higher than chance proportion of cells show *zif268* labeling during both epochs in both the adult and aged DG. The comparable number and pattern of *zif268* expression during rest is particularly striking given the profound decrease in the number of cells transcribing *zif268* during exploration in the aged DG (All data are mean ± SEM; *p < 0.05, **p < 0.01 relative to age-matched caged controls, †p < 0.05, adult vs. aged animals within the same behavioral group; ‡‡ p < 0.01 vs. random chance).

Analysis of the number of granule cells transcribing *zif268* during exploration revealed a significant main effect of condition (F_1,20_ = 25.68; p < 0.001), showing that both adult and aged animals expressed *zif268* in significantly more granule cells relative to age-matched caged control animals (Figure 6). Moreover, a trend towards a significant main effect of age (F_1,20_ = 3.40; p = 0.08), alongside a significant age by condition interaction (F_1,20_ = 4.42; p = 0.04) show that fewer granule cells transcribing *zif268* in the aged DG during exploration relative to adult animals, consistent with previous data (e.g., [7,13,25]).

#### Repeated zif268 transcription is preserved in granule cells of aged animals

In contrast to *zif268* transcribed during spatial navigation, adult and aged animals had equivalent numbers of granule cells expressing *zif268* during rest. That is, no significant differences were observed based on condition (F_1,20_ = 1.52; p = 0.23), age (F_1,20_ = 1.09; p = 0.31), or their interaction (F_1,20_ = 1.02; p = 0.32). This result is in contrast to experiment 1, in which a significant increase in the number of granule cells expressing *zif268* was observed during rest in delay animals. This may not be surprising, given the substantial variation that is seen in measures of reactivation [26]. Observing that more cells are active during rest, however, is not necessary to conclude that a pattern of *zif268* expression consistent with reactivation is occurring. It is the pattern, rather than quantity, of *zif268* expression that is the key test for this conclusion.

No significant age-related difference was observed in the probability that a granule cell that was active during rest was the same cell that was active during the previous exploration behavior. That is, the probability that a cell transcribing *zif268* during rest also transcribed *zif268* during previous behavior was greater than expected by chance for both adult (t_5_ = 9.64; p < 0.001) and aged (t_5_ = 6.12; p = 0.002) animals. Moreover, no significant age-related difference was observed in the proportion of cells expressing *zif268* during both exploration and rest, either relative to the entire granule cell population (F_1,10_ = 3.32; p = 0.11) or relative to the number of cells expected by chance (F_1,10_ = 2.32; p = 0.16).

In the aged DG, the probability that a granule cell that was active during behaviour was (a) more likely than chance to be active during subsequent rest and (b) was equally likely to be active during both episodes as granule cells in the adult DG. While the absolute probability is lower in experiment 2, it remains an order of magnitude grater than was observed in the immediate group or in the infrapyramidal blade in experiment 1. Although it remains possible that significant differences may emerge if tested in a much larger population of animals, it is clear that the expression of *zif268* in the aged DG during rest is *relatively* preserved. These data are particularly striking given the profound decrease in the number of cells transcribing *zif268* during exploration behavior in aged animals.

## Discussion

The current findings are consistent with the hypothesis that IEG expression during resting states captures experience-specific changes in neuronal activity following spatial experience. These data also support recent studies showing that granule cells maintain key physiological distinctions relative to other hippocampal regions and provide novel insights into how the behavior of these cells changes with progressive age.

The current data show that a subpopulation of granule cells in the DG are predisposed to being repeatedly active (or at least being engaged in sufficient activity to induce plasticity that coincides with *zif268* transcription) in any combination of two epochs, even in the absence of previous experience (i.e., in the immediate group). This is in striking contrast to unit recordings and IEG expression data from other hippocampal regions, both of which indicate that the probability that a single cell will be active during both rest and behavior under similar conditions is approximately equal to random chance with replacement. These data, however, are consistent with Shen et al.’s report [6] that ~50% of cells active during a single rest episode are also active during subsequent maze exploration. However, because these data were pooled from a large number of sessions after the rats were trained over several days, it is also possible that correlations between spatial behavior and preceding rest episodes reflects accumulated memories from the previous days’ experiences. In fact, such “preplay” has been previously reported in CA1 [27,28]. Based on these data, and others [16,17], it seems likely that the distribution of firing thresholds in the granule cell population is highly variable, and those cells with the lowest thresholds are predisposed to become active, both in multiple environments and between active behavior and resting states. Spatial experience, however, further increases this high “basal” correlation, making it even more likely that granule cells that are active during rest are the same cells that were active during spatial experience.

The current data show that this is also true in the aged DG – despite a decline of the number of granule cells transcribing *zif268* during exploration behavior, the proportion of cells concurrently expressing *zif268* in the foci and cytoplasm was not statistically different. This indicates that *zif268* expression during resting states in general and expression during bouts of reactivation in particular are relatively preserved in the aged DG. This dissociation between the age-related changes in *zif268* expression during rest and during exploratory behavior suggests that the source for these changes may be likewise dissociable. This notion, in turn, has implications both for the communication between CA3 and DG as well as how this pathway changes with progressive aging.

During exploratory behavior, the decrease in the number of granule cells transcribing *zif268* in aged animals, relative to adult ones, solely during spatial exploration is likely caused by decreased innervation from the perforant path. The input to the DG from the perforant path declines by approximately one-third with progressive aging [29-31]. This decreased excitatory input to the aged DG is the likely reason for the attenuation of activity-related gene expression with age. In contrast, the reactivation-related expression of *zif268* in the DG during rest is relatively preserved, and we hypothesize that this activity is mediated by the backprojection from CA3. This hypothesis is supported by the fact that both computational models (e.g., [32-34]) and experimental evidence (e.g., [34-38]) support CA3 as the most likely source of recapitulation of recent activity patterns. This is thought to stimulate DG either through direct backprojections from CA3c (nearest the hilus), or through indirect projections via mossy cells [39,40]. The current data suggest that these backprojections from CA3 to the DG are functionally intact in the aged hippocampus, and remains equally capable of inducing reactivation of recent traces in the aged DG despite a decline in granule cell activity levels during exploratory behavior in senescent animals. This hypothesis is consistent both with recent anatomical [31] and functional imaging data [7] suggesting that intrinsic connections of the hippocampus are relatively intact in aged animals. Moreover, the dissociation between resting and behaviorally-driven *zif268* expression in the aged DG is consistent with a wealth of data suggesting an increased emphasis in CA3 on the retrieval of previously stored patterns relative to input from the perforant path (see [2] for review). Although the preserved amount and pattern of *zif268* expression during rest in aged animals is consistent with the notion that encoding failure (rather than impaired consolidation *per se*) is the major cause of deficits in hippocampus-dependent behaviors in aged animals [2], this conclusion must be made with caution.

Recent studies demonstrate that the reactivation deficits that occur in aged animals are actually quite subtle. That is, when pattern reactivation is quantified on the basis of cell pair correlations, no age-related deficit is observed [41]. In contrast, when the temporal sequence of reactivation patterns across cell pairs is accounted for, a deficit emerges that correlates with memory deficits across animals [5]. It was proposed by Gerrard et al. that only sequence reactivation relies on associative synaptic plasticity, and recent data [42] showing that reactivation critically depends on co-firing of cells during behaviour within brief (50 ms) time intervals are consistent with this claim. However, precise temporal order is not required to induce long-term potentiation (LTP) in at least some hippocampal synapses [43,44], and reactivation still occurs if cells do not exhibit a temporal bias in their firing within the time required to induce spike timing-dependent plasticity [45]. These data, combined with the fact that the expression of IEGs such as *zif268* are required for enduring LTP [12], and long-term stability of place fields [46] suggests that neither *zif268* expression, nor the enduring plasticity that it mediates, require cells to fire in a strictly preserved temporal sequence. Definitive assessment of the sensitivity of *zif268* expression to the replay of specific sequences, however, would require further experiments using tasks involving more stereotyped paths of navigation. In open field exploration, as conducted here, animals take many different paths through the same physical space. Because the animal will not consistently cross the same path, cell pairs will not consistently fire in a set temporal sequence.

## Conclusions

Despite the open questions that remain, the current data show that the granule cells that express *zif268* in the DG during resting states can be predicted on the basis of recent experience. That is, using *zif268* catFISH, the majority of cells transcribing zif268 within the home cage during epoch 2 were the same cells that were active during a distinct episode of spatial experience in a different location 30 minutes earlier during epoch 1. This continued expression is consistent with memory trace reactivation in the DG, and this pattern is preserved in aged animals, despite significant declines in *zif268* expression during spatial exploration. Collectively, these data demonstrate that imaging *zif268* expression may be particularly valuable for detecting activation of neurons during resting states despite low numbers of active cells, particularly in structures with sparse activity such as the DG.

## Competing interests

The authors declare that they have no competing interests.

## Authors’ contributions

DFM designed the experiment, AG, AAA, EA, and DFM conducted the research, AG and DFM analyzed the data, and AG, EA, and DFM wrote the manuscript. All authors read and approved the final manuscript.
